# Virtual Screening, Synthesis and Biological Evaluation of *Streptococcus mutans* Mediated Biofilm Inhibitors

**DOI:** 10.3390/molecules27041455

**Published:** 2022-02-21

**Authors:** Lubna Atta, Ruqaiya Khalil, Khalid Mohammed Khan, Moatter Zehra, Faiza Saleem, Mohammad Nur-e-Alam, Zaheer Ul-Haq

**Affiliations:** 1 H. E. J. Research Institute of Chemistry, International Center for Chemical and Biological Sciences, University of Karachi, Karachi 75270, Pakistan; lubnaatta05@gmail.com (L.A.); khalid.khan@iccs.edu (K.M.K.); faizasaleem632@gmail.com (F.S.); 2 Dr. Panjwani Center for Molecular Medicine and Drug Research, International Center for Chemical and Biological Sciences, University of Karachi, Karachi 75270, Pakistan; ruqaiyakhalil@gmail.com (R.K.); moatterzehra10@yahoo.com (M.Z.); 3 Department of Clinical Pharmacy, Institute for Research and Medical Consultations (IRMC), Imam Abdulrahman Bin Faisal University, P.O. Box 31441, Dammam 31441, Saudi Arabia; 4 Department of Pharmacognosy, College of Pharmacy, King Saud University, P.O. Box 2457, Riyadh 11451, Saudi Arabia; mohnalam@ksu.edu.sa

**Keywords:** dental caries, biofilm, glucosyltransferases, virtual screening, antibiofilm, antimicrobial ADMET profiling

## Abstract

Dental caries, a global oral health concern, is a biofilm-mediated disease. Streptococcus mutans, the most prevalent oral microbiota, produces extracellular enzymes, including glycosyltransferases responsible for sucrose polymerization. In bacterial communities, the biofilm matrix confers resistance to host immune responses and antibiotics. Thus, in cases of chronic dental caries, inhibiting bacterial biofilm assembly should prevent demineralization of tooth enamel, thereby preventing tooth decay. A high throughput screening was performed in the present study to identify small molecule inhibitors of *S. mutans* glycosyltransferases. Multiple pharmacophore models were developed, validated with multiple datasets, and used for virtual screening against large chemical databases. Over 3000 drug-like hits were obtained that were analyzed to explore their binding mode. Finally, six compounds that showed good binding affinities were further analyzed for ADME (absorption, distribution, metabolism, and excretion) properties. The obtained in silico hits were evaluated for in vitro biofilm formation. The compounds displayed excellent antibiofilm activities with minimum inhibitory concentration (MIC) values of 15.26–250 µg/mL.

## 1. Introduction

Dental caries is one of the major health concerns in both developing and industrialized countries [1]. It is a biofilm-mediated disease attributed to dysbiosis in the oral microbiome [2]. The oral environment is influenced by several factors, primarily diet and host immune competence, promoting the virulence and adhesion of pathogenic microorganisms [3,4]. The prime agent associated with dental caries is *Streptococcus mutans*, which produces organic acids, exhibits acid tolerance mechanisms, and produces extracellular enzyme glucosyltransferases which produce complex sticky glucans [5]. These glucans serve as binding sites for the attachment of other microorganisms, producing a biofilm. The bacteria associated with the biofilm are resistant to host defense, mechanical and oxidative stress, and antibiotics [6]. Microbes within the mature biofilms produce copious amounts of acids, mainly lactic acid, and propionic acid, causing demineralization of the tooth surfaces and eventually dental caries [7].

Several different approaches have been developed to prevent dental caries such as mechanical methods, effective use of fluoride, and sugar substitutes (xylitol) in extreme conditions [8,9,10]. Natural products such as polyphenols, catechins, cranberry constituents, and other plant analogs exhibit antibiofilm activity [11,12,13,14]. However, the non-selective antimicrobial activity presented by these compounds results in further dysbiosis. Recently, targeted antimicrobials have been developed to eliminate *S. mutans* with a minimal effect on normal microbiota [15]. The microorganisms in the biofilm assembly have become more resistant to antibiotics and host defense systems, so there is a need for new therapeutic agents with minimal toxicity and more bioavailability.

**Figure 1:**
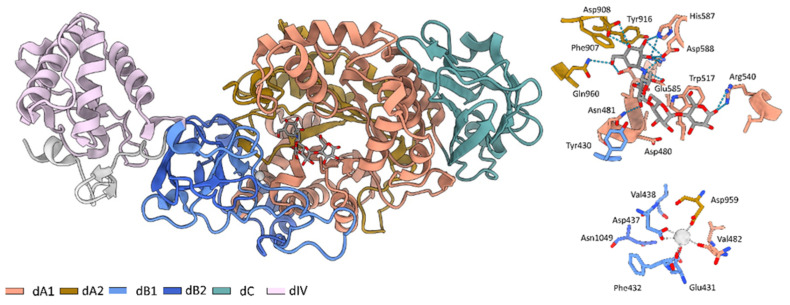
Structure of *S. mutans* glycosyltransferase (PDB: 3AIC), showing all the four domains (A, B, C and IV). Insets represents the molecular co-ordination of calcium ion (grey sphere) and acarbose (grey sticks). Hydrogen bonds between acarbose and the surrounding amino acids are presented as blue dashed lines.

The formation of dental biofilm initiates with the attachment of microorganisms on the tooth surface. The microorganism produces extracellular exopolysaccharides (EPS) which provide binding sites to other microorganisms and result in complex biofilm formation [16]. *S. mutans* is an essential contributor to the production of the extracellular matrix. It produces three types of glucosyltransferases (Gtfs): GtfB, GtfC, and GtfD. The GtfB and GtfC are mutansucrases that produce mainly insoluble glucan with (α 1–3) glycosidic linkages from sucrose. GtfD is a dextransucrase that produces soluble glucans with (α1–6) linkages. All Gtfs comprises three functional regions: N-terminal variable junction region, C-terminal glucan-binding region, and ahighly conserved catalytic region in the middle, which is essential for the glucan synthesis [17,18,19]. The crystal structure of GtfC provides crucial insights for drug design and development of the new Gtf inhibitors [20]. The secondary structure of GtfC comprises four separate domains: A, B, C, and IV (Figure 1). The ligand-binding domain lies in domain A (comprising residues of both sub-domains A1, and A2), while the calcium-binding domain is the interface of domains A and B. The presence and orientation of calcium is significant for enzymatic activity [21,22].

In the present study, pharmacophore modeling approaches, mainly ligand-based, have been implemented to identify the potential hits to inhibit biofilm formation. The multiple pharmacophore models were generated and are utilized for the screening of an in-house compound database. The molecular docking analysis of identified virtual hits was performed. The virtual hits show interactions with crucial residues of the protein. ADMET properties display a low percent of human absorption, molecular weight (250–500), and water solubility (−3.50–5.10). Further, the antibiofilm activity of the compounds was also investigated against *S. mutans* ATCC: 25175 strains. The virtual hits displayed modest antibiofilm activity against *S. mutans*.

## 2. Results and Discussion

Dental caries is a multifunctional disorder in which different oral microbiomes, predominantly *S. mutans*, interact with dietary sugars to produce cariogenic plaques. The available treatments to combat dental caries are non-selective, board-spectrum, and cause dysbiosis, which in severe cases lead to oral cancers. The present study was carried out to identify, synthesize, and characterize novel small molecule selective inhibitors targeting *S. mutans* glucosyltransferase.

### 2.1. Virtual Screening

**Figure 2:**
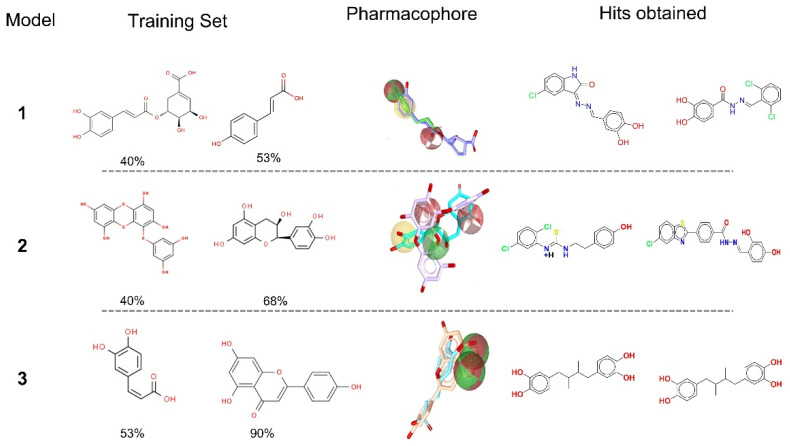
Chemical structures of the training set employed for pharmacophore modelling, and the top-ranked hits from each model.

For virtual screening, a series of ligand-based pharmacophore models were developed using the features of the training set. The models were developed using different combinations of shared pharmacophoric features of the training compounds i.e., 5-o-caffeoyl shikimic acid and p-coumaric acid (model 1), eckol and epicatechin (model 2), and apigenin and caffeic acid (model 3). The three models presented different pharmacophoric features, resulting in scaffold hopping and chemical diversity. For instance, model 1 comprises four features: two hydrogen bond acceptors (HBA) and one hydrogen bond donor (HBD), and one hydrophobic (Figure 2). Model 2 exhibits four features: one hydrophobic, two hydrogen bond acceptors, and one hydrogen bond donor. In the case of model 3, there were no hydrophobic characteristics, and the selected features included two hydrogen bond donors and two hydrogen bond acceptors.

The three selected models were subjected to vigorous testing using a dataset of reported inhibitors, negative controls, and decoys. Further, the sensitivity and selectivity of the models were accessed. The sensitivity and specificity of model 1, model 2, and model 3 was 0.6, 0.7, and 0.7, respectively (Table 1). The retrieval rate of the active compounds from the training datasets was quite encouraging, so these optimized models were recruited for the screening of an in-house dataset of drug-like compounds.

Model 1, model 2, and model 3 retrieved 2043, 1730 and 2180 hits, respectively. In total, 5953 virtual hits were obtained from these multiple pharmacophore models. To find the possible drug candidates, Lipinski’s rule of five (Ro5) was applied, which resulted in 3059 unique drug-like hits. The pharmacophore-based virtual screening resulted in 3059 unique hits. The resultant hits were then subjected to docking studies using the crystal structure of *S. mutans* glycosyltransferase. Prior to the docking studies, the benchmarking of the docking software was performed. Based on the RMSD values between the coordinates of the cognate ligand and the simulated pose (Table S2 Supplementary Materials), MOE-dock was selected for the docking of the identified virtual hits from the preceding step.

For the identification of potential drug like compounds, PLIF analysis was performed after molecular docking to analyze the interaction patterns of the compounds with the active site of glucosyltransferases (Figure S1 Supplementary Materials). The active site of glycosyltransferase comprises several charged and polar residues, including Asp480, Glu515, Trp517, His587, Asp588, Asp593, Asn862, Asp909, and Asn914. The complementarity of the active site is reflected in the presence of polar and heterocycles in the identified hits. The scaffolds selected by models 1 and 2 include different classes of heterocyclic compounds indole and hydrazide (A3898, A3566, A4554, and A6996). Model 3 identified two hits, A13419 and A4554, a carbothioamide and indole, respectively. The final six compounds were selected based on their good interaction with the crucial residues of protein. The 2D structure of the selected hits (*n* = 6) and the interaction pattern in static mode are presented in Figure 2 and Table 2, respectively.

**Figure 3:**
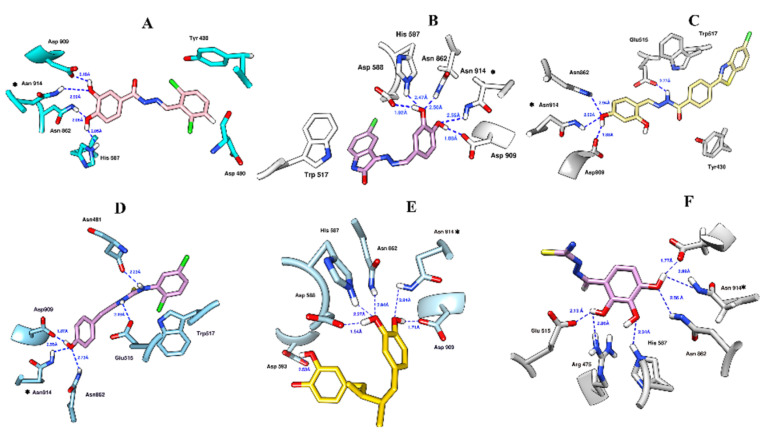
The 3D interactions of the selected hits (**A**) A3898 (pink), (**B**) A3566 (purple), (**C**) A6996 (yellow), (**D**) A4554 (purple), (**E**) A12324 (gold), (**F**) A13419 (plum).

All six hits exhibited hydrogen bonds with Asn862 and Asn914 in the active site. Further, A3898 also mediated hydrophobic interactions with Tyr430 and Asp480 (Figure 3A). A3566 extends hydrophobic contacts with the indole ring of Trp517 and His587 (Figure 3B). Hit A6996 shows hydrophobic interaction with Tyr430 and Trp517. Moreover, A6996 establishes a hydrogen bond with the carbonyl moiety of Asp909. Nitrogen moiety in the aliphatic chain forms a hydrogen bond with Glu515 (Figure 3C). The amide moiety of A4554 is involved in hydrogen bond interactions with Asn481 and Glu515. The compound also shows hydrophobic interactions with Trp517 (Figure 3D). The hits obtained from model 3 include A13419 and A12324. A12324 exhibits hydrogen bonds with His587, Asp588, and Asp593 (Figure 3E). A13419 also forms a hydrogen bond with Arg475, Glu515, His587, and Asp909 (Figure 3F).

### 2.2. ADMET Profiling

**Figure 4:**
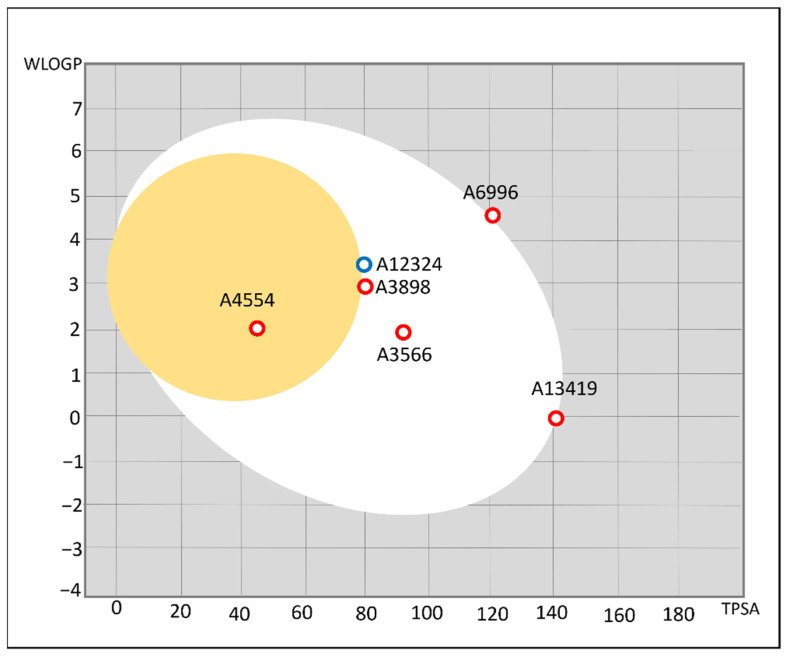
Predicted human intestinal (HIA), and P-glycoprotein efflux (P-gp) inhibition of the selected hits. The compounds lying in the egg-white region could be absorbed by the human intestinal lumen (HIA permeable), and those in the yolk can also permeate the blood–brain barrier. The compounds with blue spots are substrates of P-glycoprotein, while non-substrates are presented as red spots. The visual was obtained from SwissADME webserver.

Pharmacokinetic profiles of selected hits were predicted by SwissADME. The molecular weight of the selected hit varied from 227–423 (g/mol), Plog values were in the range of 1.22–3.01 and water solubility varied from −1.43–5.74, respectively (Table 2). The compounds A12324, A38989, and A3566 were found to exhibit decent oral bioavailability (Table 2 and Figure 4). A4554 could permeate the blood–brain barrier, which is not desirable in the current context. Except for A12324 (a substrate of PGP, PGP+), none of the compounds are P-glycoprotein (PGP) binders, that is, the intestinal absorption of these compounds is not compromised by the activity of p-glycoprotein efflux pumps located in the intestinal lumina. Further, Protox-II server was employed to evaluate organ toxicity (hepatotoxicity) and oral toxicity of the selected hits. All the compounds were found to be non-cytotoxic and safe for oral consumption.

### 2.3. Antimicrobial and Biofilm Inhibition

The selected compounds were then evaluated for the antibiofilm activity against *S. mutans* strains (ATCC: 25175). All compounds displayed modest biofilm activity against *S. mutans*. The compound A3566 showed the most promising antimicrobial and antibiofilm properties against the targeted pathogen with MIC of 15.62 μg/mL (Table 2).

**Figure 5:**
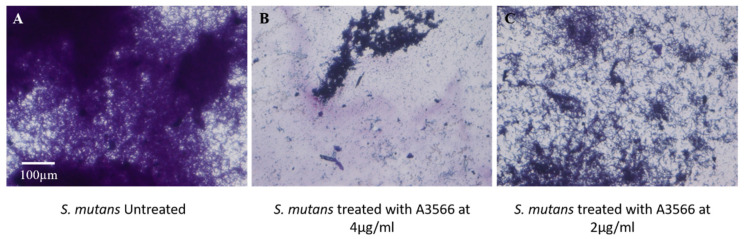
Biofilm inhibition potential of LN 3566 against *S. mutans*. (**A**) Untreated control, (**B**) treated with A3566 at 4 µg/mL, (**C**) treated with LN 3566 at 2 µg/mL. Images were captured using a Nikon TE2000 inverted microscope with 200× total magnification.

The biofilm inhibition observed in the crystal violet assay was further consolidated by light microscopy analysis. The untreated well of *S. mutans* showed intact biofilm densely stained with crystal violet (Figure 5A). When *S. mutans* was treated with A3566 at MIC (4 µg/mL) concentration, the biofilm formation was reduced to almost 80%, whereas, at sub-MIC (2 µg/mL) level almost 50% of the biofilm was reduced (Figure 5B,C). The microscopic evaluation further complemented the biofilm-inhibiting potential of A3566 against *S. mutans*.

## 3. Materials and Methods

### 3.1. Pharmacophore-Based Virtual Screening

We curated a database of compounds with inhibitory activity reported against glycosyltransferases through literature searches. The curated database consisted of structurally diverse chemical scaffolds (Table S1 Supplementary Materials) including quinazoline, flavonoids, and polyphenols [11,18,23,24]. To validate the hypothesis, and to reduce the instances of false-positive hits, a database of reported inactive compounds [25,26] (*n* = 9, Table S2 Supplementary Materials), and a decoy database was also curated. A total of 1695 decoys were generated using DUDE web server [27]. For lead identification, an in-house dataset comprising 14,500 compounds of both synthetic and natural origins was used.

All the chemical structures (excluding decoys) were sketched using ChemDraw Ultra Bio version 9.0 [28]. In the case of decoys, the chemical structures were obtained from the DUDE web server in SDF format which was converted to MOL2 format using Obabel [29]. The compound libraries were then exported to MOE 2019 [30] software to assign partial charges chemical ionization, protonation, the addition of missing hydrogen atoms using Protonate 3D algorithm [31] followed by minimization under MMFF94x force field [32] with a gradient value of 0.1 kcal/molA^2^.

### 3.2. Pharmacophore Generation and Validation

The pharmacophore models were constructed using LigandScout 4.4 [33]. For every compound in the training set, thirty different conformations were generated using OMEGA [34]. Different functional groups of the compounds were recruited to generate a series of pharmacophore models mapping the shared features of the training set. Different combinations of compounds were aligned to generate shared feature pharmacophore. The developed models were then subjected to screening for initial validation. The sensitivity and selectivity of different models were then assessed. The models with optimum matrices were employed to screen the database.

### 3.3. Preparation and Screening of Database

For hit identification, we employed an in-house database comprising 14,500 diverse compounds, of both natural and synthetic origin. All compounds were prepared for screening using the protocol mentioned above. For virtual screening, the ‘idbgen’ in LigandScout was used to convert the database into LigandScout specific multi-conformation database (ldb), which can store multiple conformations of each compound. After the removal of duplicates, the databases were screened with generated pharmacophore models to identify the potential hits. The identified virtual hits were filtered with the Lipinski rule of five [35] to identify the drug-like compounds. The potential hits obtained after the Lipinski filter were analyzed for their binding mode with molecular docking.

### 3.4. Molecular Docking

The binding energy of each compound was calculated using molecular docking [36,37]. Initially, a redocking experiment was conducted to establish the efficiency of the docking protocol to reproduce the crystal pose. Coordinates of glucansucrase Streptococcus mutans were obtained from the RSCB protein data bank (PDB ID:3AIC) [38]. The structure was imported in MOE and was subjected to the ‘protein preparation’ module for the addition of missing atoms, assignment of partial charges (using AMBER99), and protonation (using Protonate 3D algorithm). For ligand placement and scoring, the triangle matcher algorithm and London-dG scoring function were used. The docking site was designated using the coordinates of the cognate ligands (AC1 and GLC). For each compound, a single top-ranked pose was retrieved, and binding energy was recorded.

Furthermore, to analyze the protein–ligand contact profile, the protein–ligand interaction fingerprint (PLIF) module in MOE was used. PLIP [39] analysis helps to identify the key residues of the respective protein which was responsible for binding with the ligand. PLIP provides detail of seven different interactions (hydrogen bonds, hydrophobic contacts, pi-stacking, pi-cation interactions, salt bridges, water bridges, and halogen bonds. The compounds exhibiting contacts with crucial residues were selected for hit optimization.

### 3.5. ADMET Profiling

The ADMET profiles of the identified compounds were evaluated with the help of the SwissADME web server [40,41].

### 3.6. General Procedure for the Synthesis of Compounds

The identified hits were then synthesized to perform the biochemical assays against the biofilm.

A3566: In the first step, hydrazone was prepared by refluxing a mixture of 5-chloroisatin (1 g) and hydrazine hydrate (10 mL). Then, hydrazone so prepared (1 mmol) and 3,4-dihydroxybenzaldehyde (1 mmol) in methanol were reacted together under reflux for 3 h. TLC was used for monitoring the proceeding of reaction. After reaction completion, crystalline powder of Schiff base was collected, washed with methanol, and dried. Recrystallization from methanol afforded pure crystals. The structure was confirmed by using ^1^HNMR, and EI-MS.

Yield: 69%; ^1^H NMR: (300 MHz, DMSO-*d*_6_): 10.9 (s, 1H, N–H), 9.6 (s, 1H, O–H), 8.57 (s, 1H, –N=CH), 7.4 (s, 1H, H-4), 7.38 (d, 1H, *J*_6,7_ = 7.5 Hz, H-6), 7.25 (d, 1H, *J*_6′,5′_ = 7.8 Hz, H-6′), 7.1 (s, 1H, H-2′), 6.9 (d, 1H, *J*_5′,6′_ = 7.8 Hz, H-5′), 6.86 (d, 1H, *J*_7,6_ = 7.5 Hz, H-7); EI MS: *m*/*z* (rel. abund.%), 315 (M^+^, 5), 287 (100), 180 (32), 152 (85), 109 (36), 63 (40).

A3898: (E)-N′-(2,6-dichlorobenzylidene)-3,4-dihydroxybenzohydrazide was synthesized by treating an equimolar amount of 2,6-dichlorobenzaldehyde with 3,4-dihydroxybenzohydrazide in a suitable amount of DMF and stirring the reaction mixture overnight at room temperature. Progress of the reaction was monitored by thin-layer chromatography (TLC). After completion, the reaction mixture was poured in ice water to precipitate out the final product, which was filtered and washed with hot ethanol to afford the pure product. The compound was characterized by ^1^HNMR and EI-MS analysis.

Yield: 97%; ^1^H-NMR (300 MHz, DMSO-*d*_6_); 11.83 (s, 1H, NH), 9.27 (s, 2H, OH), 8.59 (s, 1H, N=C-H), 7.56 (d, 2H, *J*_3′,4′-5′-4__′_ = 6 Hz, H-3′, H-5′), 7.45 (dd, 1H, *J*_4′-3′-4′-5′_ = 7.5 Hz, H-4′), 7.39 (dd, 2H, *J*_6,2–2,6_ = 1.8 Hz, H-6, H-2), 6.82 (d, 1H, *J*_5,6_ = 8.1 Hz, H-5); EI MS *m*/*z* (% rel. Abund.); 324 (M^+^, 90), 256 (57), 152 (47), 136 (100), 120 (65), 109 (58), 81 (24), 44 (11).

A4554: In the first step, 2,3,4-trihydroxy benzaldehyde (10 mmol) and thiosemicarbazide (10 mmol) were taken in ethanol (50 mL) into a 250 mL round-bottomed flask with a few drops of glacial acetic acid. The reaction mixture was refluxed for 4 h. Precipitates of the product appeared in the reaction mixture. Progress of the reaction was monitored by thin-layer chromatography (TLC). After completion, precipitates were filtered and washed with 10 mL cold ethanol to afford the pure product in good yield. In the second step, thiosemicarbazone intermediate (0.5 mmol), 3,4-dichlorophenacyl bromide (0.5 mmol), and triethylamine (0.5 mmol) were taken in ethanol into a 100 mL round-bottomed flask and refluxed for 3 h. Progress of the reaction was monitored by the thin layer chromatography (TLC). Precipitates appeared in the reaction mixture which was filtered and washed with 5 mL cold ethanol to afford the pure products. The compound was characterized by spectroscopic analysis.

Yield: 75%; ^1^H-NMR (400 MHz, DMSO-*d*_6_): δ 11.99 (s, 1H, NH), 9.63 (s, 1H, OH), 9.46 (s, 1H, OH), 8.48 (s, 1H, OH), 8.18 (s, 1H, H-C=N), 8.065 (d, *J*_2__″__,6__″_ = 1.6Hz, 1H, H-2″), 7.83 (dd, *J*_6__″__,2__″_ = 1.6 Hz, *J*_6__″__,5__″_ = 8.4 Hz, 1H, H-6″), 7.66 (d, *J*_5__″__,6__″_ = 8.4 Hz, 1H, H-5″), 7.50 (s, 1H, H-5′), 6.88 (d, *J*_6,5_ = 8.8 Hz, 1H, H-6), 6.39 (d, *J*_5,6_ = 8.4 Hz, 1H, H-5); EI MS: *m*/*z* (rel. abund.%); 394 (M^+^, 100), 396 (M^+2^, 64), 324 (17), 285 (35), 190 (28), 81 (24).

A6996: Synthesis and characterization of the compound published previously [42].

A13419: 2,3,4-Trihydroxy benzaldehyde (1 mmol) and thiosemicarbazide (1 mmol) were taken in ethanol (5 mL) into a 100 mL round-bottomed flask and acidified with a few drops of glacial acetic acid. The reaction mixture was refluxed for 4 h. Precipitates of the product appeared in the reaction mixture. Progress of the reaction was monitored by thin-layer chromatography (TLC). After completion, precipitates were filtered and washed with 10 mL cold ethanol to afford the pure (E)-2-(2,3,4-trihydroxybenzylidene)hydrazine-1-carbothioamide product in good yield.

Yield 78%; ^1^H-NMR (300 MHz, DMSO-*d*_6_): 11.16 (s, 1H, NH), 9.48 (br s, 1H, OH), 8.94 (br s, 1H, OH), 8.40 (br s, 1H, NH2), 8.20 (s, 1H, –HC=N–), 7.93 (br s, 1H, OH), 7.72 (br s, 1H, NH2), 7.11 (d, 1H, *J* = 8.4 Hz, phH), 6.32 (d, 1H, *J* = 8.4 Hz, phH). MS (ESI): *m*/*z* (rel. abund.%), 228 (M^+1^)

A12334 [43]: This is a commercially available compound and was obtained from a local market.

### 3.7. Antimicrobial and Biofilm Inhibition Studies

Streptococcus mutans (ATCC: 25175) strains were used for in vitro assessment of antimicrobial and antibiofilm activities of the hit compounds. *S. mutans* was grown and passed using heart infusion broth (Oxoid, UK) supplemented with 1% sucrose. The strains were grown aerobically at 37 °C for 24 h.

The minimum inhibitory concentration of the selected compounds was evaluated using the micro broth dilution method as described previously [44]. Briefly, compounds were two-fold serially diluted in the range of 500 to 1 µg/mL. *S. mutans* (5 × 105 cells/mL) were inoculated in each except negative control. The eleventh and twelfth wells served as positive (media plus *S. mutans*) and negative (only media) controls. The plate was incubated overnight at 37 °C. The following day, the lowest concentration of the compound inhibiting bacterial growth was recorded as the minimum inhibitory concentration (MIC). The minimum bactericidal concentration (MBC) was evaluated by plating the optically clear well to a new fresh media plate. The lowest concentration that inhibited bacterial growth was recorded as MBC. The biofilm-inhibiting potential of the compounds was evaluated using the crystal violet staining method [45]. The following equation is used for the calculation of % biofilm inhibition.

%biofilm inhibition = {(O.D. in control − O.D. of test)/O.D. in control} × 100

The biofilm inhibition was further complemented with light microscopic observation after staining with crystal violet as described previously [44]. Briefly, *S. mutans* was treated with A3566 at inhibitory and sub-inhibitory concentrations. After incubation, the wells were washed and heat-fixed. Biofilm mass was stained with 0.1% crystal violet for 20 min. After staining, the excess dye was removed and plates were dried. Images of biofilm structure were visualized under Nikon TE2000 inverted microscope (Nikon, Tokyo, Japan) at 200× total magnification.

## 4. Conclusions

In this study, in silico strategies and microbiological assays/bioassays were employed to identify small molecules that target glycosyltransferases from *S mutans*. A virtual screening based on pharmacophores followed by intermolecular interaction profiling produced six hits. These hits exhibited high binding affinity, due to a combination of hydrophobic interactions and electrostatic interactions with key target residues. Additionally, these compounds displayed excellent antibiofilm activity when tested due to their good pharmacokinetic profile as predicted by SwissADME. The results highlighted the potential of the identified hits as candidates for in vivo testing, which in the future may serve as the basis for the development of new, effective, and potent derivatives for the treatment of dental caries.

## 5. Prospects and Limitations

As a result of the limited number and diversity of compounds reported in the study, one of its intrinsic limitations is the small training set. Thus, the pharmacophore hits may not represent the full range of possible ligands with similar properties. Another limitation comes from the adoption of the antibacterial and bio-film inhibition assay as a surrogate for in vitro glycosyltransferase inhibitory assay. Future studies employing larger datasets and more exhaustive methods are required to explore available chemical space and identify potent inhibitors of *S. mutans* glycosyltransferase.

## Figures and Tables

**Table 1:** Validation hit rate of the generated pharmacophore models.

Model	Actives	In-Actives	Decoys	Sensitivity
Model 1 (5-*O*-caffeoyl shikimic acid + *p*-coumaric acid)	16	2	100	0.6
Model 2 (Eckol + Epicatechin)	17	1	81	0.7
Model 3 (Apigenin and Caffeic acid)	16	1	75	0.7

**Table 2:** ADMET properties and results of the microbiological assays of the selected hits.

Code	ADMET Properties				Biological Activity			
	Molecular Weight (g/mol)	Lipophilicity (Qplog Po/W)	Water Solubility (PlogS)	Oral Absorption	MIC ^1^ (μg/mL)	MBC ^2^ (μg/mL)	MBIC ^3^ (μg/mL)	Biofilm Inhibition (%)
A3566	315	1.86	−3.68	Low	15.62	n.d.	3.91	71.86
A3898	325	1.95	−4.6	High	250.00	250.00	250.00	86.50
A4554	341	3.01	−5.09	High	125.00	n.d.	125.00	87.55
A6996	423	2.94	−5.74	Low	-	-	-	-
A12324	302	2.38	−4.5	High	250.00	n.d.	250.00	88.58
A13419	227	1.22	−1.43	Low	250.00	n.d.	250.00	88.57

^1^ Minimum Inhibitory Concentration, ^2^ Minimum Bactericidal Concentration, ^3^ Minimum Biofilm Inhibitory Concentration, n.d. Not Determined.

## Data Availability

Data are available from authors on request.

## Supplementary Materials

The following supporting information can be downloaded online. Table S1: Training dataset collected from literature, Table S2: Structures of the inactive compounds obtained from literature, Table S3: Benchmarking results using RMSD between simulated and cognate poses, and Table S4: Results of the cytotoxicity prediction of the compounds using Pro Tox -11 webserver, Figure S1: PLIF analysis of the virtual hits.

## Sample Availability

Samples of the compounds are available from the authors.
