# Supplementary material for: Virtual Screening, Synthesis and Biological Evaluation of Streptococcus mutans Mediated Biofilm Inhibitors
Source: Molecules. 2022 Feb 21;27(4):1455. doi: 10.3390/molecules27041455 (PMC8876203; doi:10.3390/molecules27041455)
Supplement: Supplementary file 1 [file molecules-27-01455-s001.zip › molecules-1561158-supplementary.pdf]

Table S1. Training Dataset collected from literature

S.No	***COMPOUND***	***STRUCTURE***	***% INHIBITION***
1	(E)-5-((3-(3,4-dihydroxyphenyl)acryloyl)oxy)-3,4-dihydroxycyclohex-1-ene-1-carboxylic acid	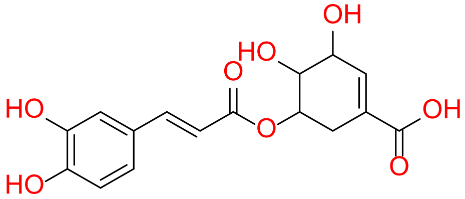	40
2	(E)-3-(hydroxy(phenyl)methylene)-6,6-dimethyl-1,5,7-tris(3-methylbut-2-en-1-yl)bicyclo[3.3.1]nonane-2,4,9-trione	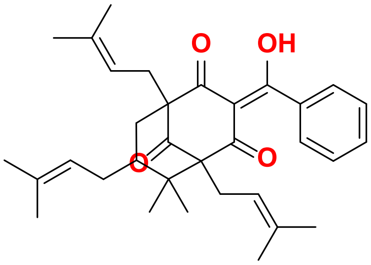	48
3	(E)-3-(3,4-dihydroxyphenyl)acrylic acid	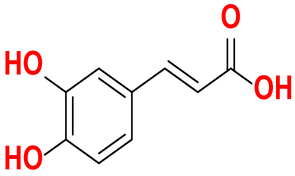	53.1
4	2-(3,4-dihydroxyphenyl)chromane-3,5,7-triol	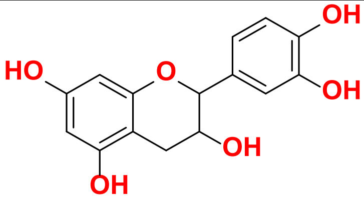	45.8
5	(E)-3-((3-(3,4-dihydroxyphenyl)acryloyl)oxy)-1,4,5-trihydroxycyclohexane-1-carboxylic acid	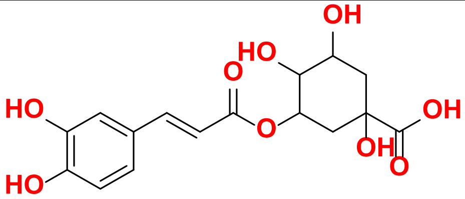	40
6	4-(3,5-dihydroxyphenoxy)dibenzo[b,e][1,4]dioxine-1,3,6,8-tetraol	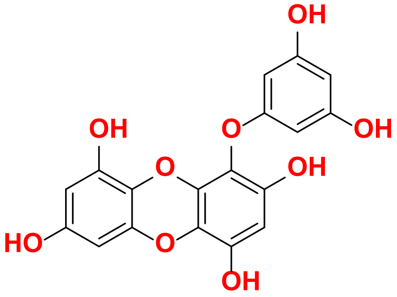	67.8

7	2-(3,4-dihydroxyphenyl)chromane-3,5,7-triol	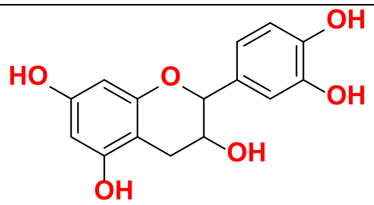	40
8	5-methyl-6-(((3,4,5-trimethoxyphenyl)amino)methyl)quinazoline-2,4-diamine	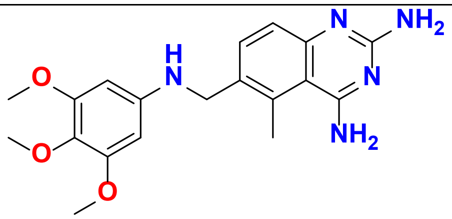	49.08
9	(E)-3-(4-hydroxyphenyl)acrylic acid	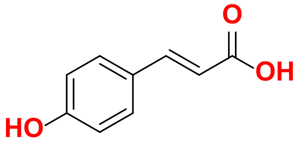	76
10	3-((3,4-dihydroxy-5-(hydroxymethyl)tetrahydrofuran-2-yl)oxy)-2-(3,4-dihydroxyphenyl)-5,7-dihydroxy-4H-chromen-4-one	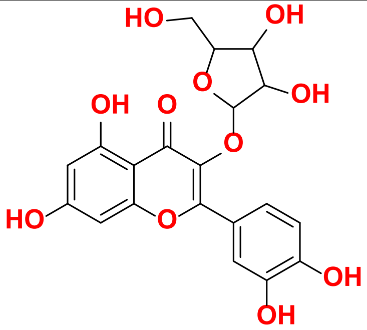	45
11	2-(3,4-dihydroxyphenyl)-5,7-dihydroxy-3-((3,4,5-trihydroxy-6-methyltetrahydro-2H-pyran-2-yl)oxy)-4H-chromen-4-one	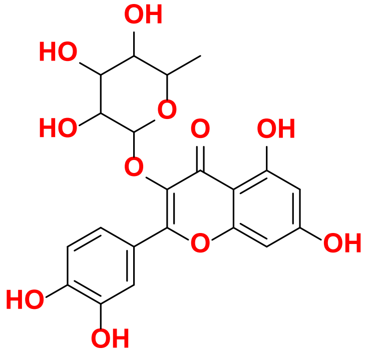	28
12	3,5,7-trihydroxy-2-(4-hydroxy-3-((3,4,5-trihydroxy-6-(hydroxymethyl)tetrahydro-2H-pyran-2-yl)oxy)phenyl)-4H-chromen-4-one	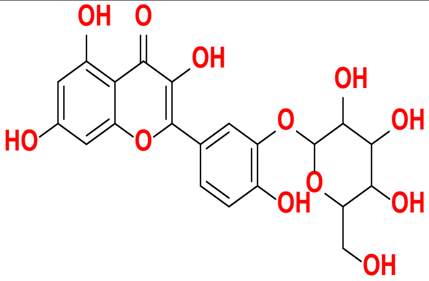	15

13	2-(3,4-dihydroxyphenyl)-5-hydroxy-3,7-bis((3,4,5-trihydroxy-6-(hydroxymethyl)tetrahydro-2H-pyran-2-yl)oxy)-4H-chromen-4-one	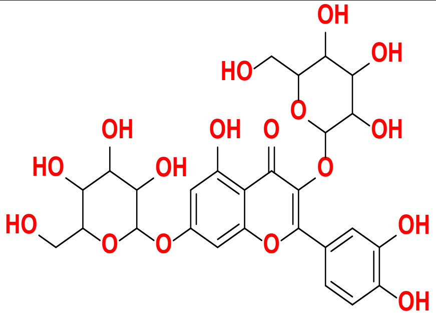	16
14	3,5,7-trihydroxy-2-(3,4,5-trihydroxyphenyl)-4H-chromen-4-one	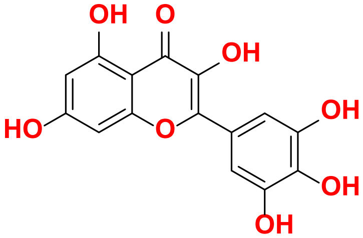	28
15	5,7-dihydroxy-2-(4-hydroxyphenyl)-4H-chromen-4-one	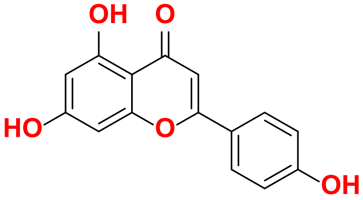	90
16	5,6,7-trihydroxy-2-phenyl-4H-chromen-4-one	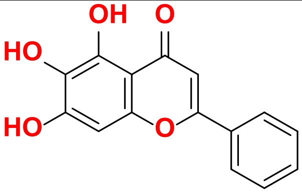	50.1
17	2-(3,4-dihydroxyphenyl)-3,5,7-trihydroxy-4H-chromen-4-one	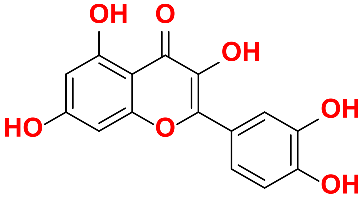	11-14

Table S2. The inactive compounds from literature

S.No	IUPAC NAME	STRUCTURE
1.	(2S,3R,4R,5S,6R)-2-(3-((E)-4-hydroxy-3-methoxystyryl)phenyl)-6-(hydroxymethyl)tetrahydro-2H-pyran-3,4,5-triol	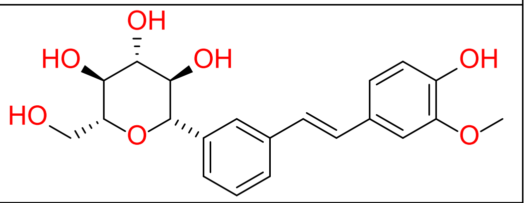
2.	4,4'-(ethane-1,2-diyl)dianiline	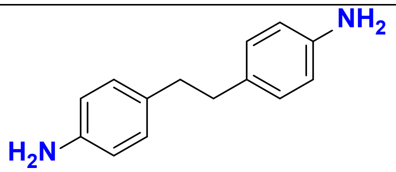
3.	1,2 diphenyl ethane	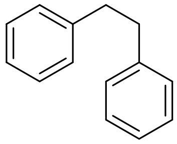
4.	(E)-1.2-diphenylethene	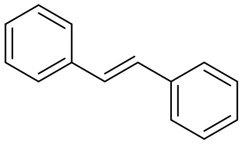
5.	(E)-1-(2-4-dihydroxyphenyl)-3-phenylprop-2-en-1-one	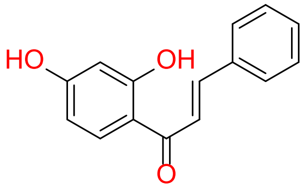
6.	(E)-1-(2,4-dihydroxyphenyl)-3-(2-hydroxyphenyl)prop-2-en-1-one)	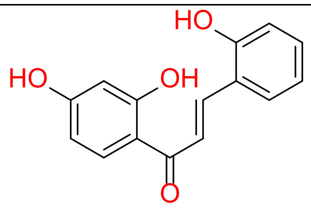
7.	(E)-3-(2-hydroxyphenyl)-1-(3-hydroxyphenyl)prop-2-en-1-one	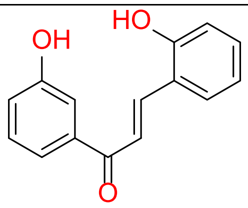
8.	(E)-1-(3,5-bis(dimethylamino)-4-hydroxyphenyl)-3-(4-methoxyphenyl)prop-2-en-1-one	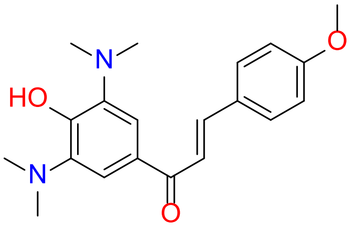

9.	(E)-1-(3,4-dimethoxyphenyl)-3-(3-hydroxy-4-methoxyphenyl)prop-2-en-1-one	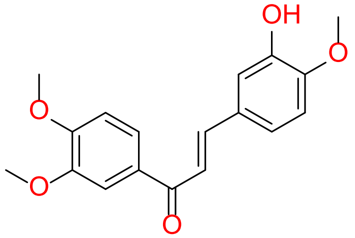

Table S3. Benchmarking of MOE and Autodock software.

Software	Number of poses	RMSD (Å)	Score (kcal/mol)
MOE	1	1.56	-8.60
	2	1.57	-8.50
	3	2.27	-8.90
	4	2.37	-8.60
	5	4.80	-9.04
Autodock	1	2.52	-8.60
	2	2.78	-8.50
	3	3.27	-8.30
	4	3.47	-8.20
	5	3.86	-8.20

Table S4. Results of the cytotoxicity prediction of the compounds using Pro Tox -11 webserver.

Compounds	Prediction	Probability
A3566	Inactive	0.57
A3989	Inactive	0.67
A4554	Inactive	0.69
A6996	Inactive	0.59
A12324	Inactive	0.77
A13419	Inactive	0.56

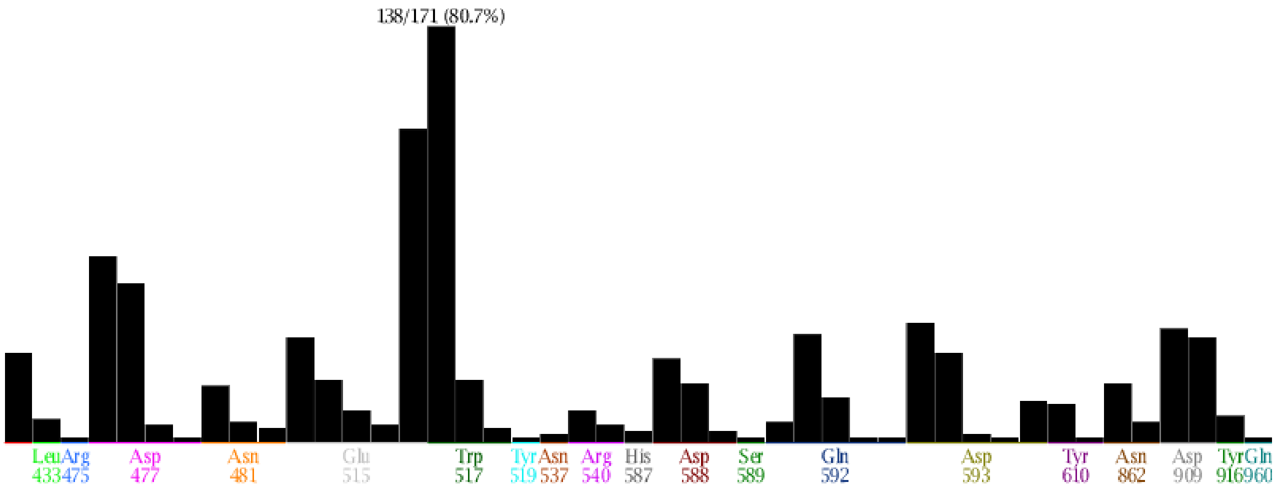

Figure S1. PLIF analysis of the virtual hits.
